# JAK2, CALR, and MPL Mutation Profiles in BCR-ABL Negative Myeloproliferative Neoplasms, a Referral Center Experience in the Middle East

**DOI:** 10.30699/IJP.2021.136458.2495

**Published:** 2021-01-24

**Authors:** Moeinadin Safavi, Ahmad Monabati, Akbar Safaei, Maryam Sadat Mirtalebi, Masoumeh Faghih

**Affiliations:** 1 *Molecular Pathology and Cytogenetic Section, Department of Pathology, School of Medicine, Tehran University of Medical Sciences, Tehran, Iran*; 2 *Molecular Pathology and Cytogenetic Section, Department of Pathology, School of Medicine, Shiraz University of Medical Sciences, Shiraz, Iran*; 3 *Hematology Research Center, Shiraz University of Medical Sciences, Shiraz, Iran*

**Keywords:** CALR JAK2 MPL MPN

## Abstract

**Background & Objective:**

JAK2, CALR, and MPL genes play pivotal roles in the pathogenesis of BCR-ABL negative myeloproliferative neoplasms. This study was conducted to evaluate the frequency of JAK2, CALR, and MPL mutations in BCR-ABL negative myeloproliferative neoplasms and their association with demographic data and hematologic parameters in a referral center, in the Middle East.

**Methods:**

Seventy-one patients with BCR-ABL negative myeloproliferative neoplasms were evaluated for JAK2 V617F, CALR type 1, CALR type 2, and MPL by allele-specific PCR and conventional PCR from 2018 to 2019.

**Results:**

Twenty-three patients were categorized as polycythemia vera, JAK2 V617F was observed in 91.3% of these cases. Thirty-eight patients were classified as essential thrombocythemia of which 52.6% showed JAK2 V617F, 18.4% demonstrated CALR type 1, 7.9% denoted CALR type 2 and there was no mutation reported in 21.1%. Seven patients were recognized as primary myelofibrosis and exhibited JAK2 V617F mutation in 57.1%, CALR type 1 in 14.3 %, CALR type 2 in 14.3% and no mutation in 14.3%. Three patients were diagnosed as MPN, unclassifiable and 33.3% revealed JAK2 V617F mutation, and no mutation was found in 66.6%. The age (59.15±13.10) and neutrophil percent (70.78±10.14) were higher in patients with JAK2 V617 mutation compared to other mutations (*P*=0.000, and *P*=0.03). Platelet count was significantly higher in patients with CALR type 1 mutation (1240400± 402053) (*P*=0.000).

**Conclusion:**

JAK2 V617F was associated with patients’ higher age and higher neutrophil count in CBC. CALR mutation had an association with higher platelet count. No MPL mutation was found in this study and it seems that its frequency is lower than what is expected in this region.

## Introduction

Myeloproliferative neoplasms (MPN) are characterized by clonal myeloid cell proliferation, bone marrow fibrosis, and peripheral blood abnormalities. The World Health Organization (WHO) provides diagnostic criteria for BCR-ABL negative MPNs, including polycythemia vera (PV), essential thrombocythemia (ET), primary myelofibrosis (PMF), chronic eosinophilic leukemia (CEL), chronic neutrophilic leukemia (CNL), and myeloproliferative neoplasms, unclassifiable (MPN-U) (1).

Common molecular disorders in MPN include mutations in the JAK2, MPL, and CALR gene. JAK2 V617F mutation was discovered as a driver mutation in MPN patients in 2005 and became a research hotspot since then. The JAK2 gene produces the Janus kinase 2 protein that takes part in the JAK-STAT signaling pathway and affects cellular proliferation and differentiation. Exon 10 mutations in the MPL gene are used as one of the diagnostic criteria. MPL is the receptor of thrombopoietin and has a pivotal role in megakaryopoiesis and platelet production (1-5). Recently, frameshift mutations related to exon 9 of the CALR gene using next-generation sequencing have been found in patients with ET and PMF who do not have the MPL or JAK2 mutation. CALR is a multi-task protein with several roles including calcium equilibrium regulation, cell proliferation, differentiation, and apoptosis (5-7).

Due to the different frequency of JAK2, MPL, CALR mutations and the difference in the course of myeloproliferative neoplasms with different mutations and considering that a comprehensive study has not been established in the Iranian population about myeloproliferative neoplasms and these mutations so far, this study was conducted in a referral center in the southwest of Iran, the Middle East.

## Materials and Methods

Seventy-one patients with BCR-ABL negative myeloproliferative neoplasms whose information was registered in the molecular pathology department of Shiraz Medical School, the southwest of Iran, were included in this study from 2018 to 2019. Patients’ demographic data (like age and sex) and hematologic characteristics of myeloproliferative neoplasms (like hemoglobin level, leukocytosis, and platelet count) were extracted from the database registered in the department of molecular pathology.

The presence of JAK2, CALR and MPL gene mutations was detected by allele-specific PCR and conventional PCR. An allele-specific PCR was applied for detection of JAK2 V617F mutation using two forward primers and a reverse primer with the following sequences: Forward (specific):5′AGCATTTGGTTTT AAATTATGGAGTATATT3′, Forward (internal control): 5′ATCTATAGTCATGCTGAAAGTAGGAGAAAG3′, Reverse:5′CTGAATAGTCCTACAGTGTTTTCAGTTTCA3′. Five μL of patient's DNA was amplified by 1 μL of reverse primer and 0·5 μL of each forward primers at an annealing temperature of 58°C during 35 cycles of PCR. The final product was run on a 2% agarose gel and a 203-bp band was considered as a positive result and the 218 bp band was considered as the internal control (8).

CALR type 1 and type 2 mutations were evaluated by primers targeting exon 9. PCR primer sets included Forward1 5´-GCAGCAGAGAAACAAATGAAGG-3´, Forward2 5´-GCAGAGGACAATTGTCGGA-3´, and Reverse (reverse primer) 5´-AGAGTGGAGGAGGGGAAC AA-3´. Five μL of DNA template, 1 μL (10 pmol) of each forward primer and 1 μL of reverse primer were added to 12.5 μL PCR master mix (final volume of 25 μL). An initial denaturation at 94°C for 2 min was followed by 40 cycles of denaturation at 94°C for 30 sec, annealing at 64°C for 30 sec, and extension at 72°C for 30 sec and eventually followed by a single round of final extension at 72°C for 5 min. Product sizes of 302 bp and 272 bp after electrophoresis on 2% agarose gel and UV transillumination were considered CALR type1 and type 2 mutations (9).

An allele-specific PCR was used for the detection of MPLW515K/L point mutations. The two reverse primers were specific for MPL mutations (W515L primer: 5′-CCTGTAGTGTGCAGGAAACTGCA-3′; W515K primer: 5′- CCTGTAGTGTGCAGGA AACTGCTT-3′). The outer primer pairs (forward primer: 5′-TAGGGGCTGGCTGGATGAG-3′; reverse primer: 5′-CTTCGGCTCCACCTGGTCC-3′) were utilized as the internal control. Five μL of DNA template, 1 μL of each outer primers and 0.5 μL of each reverse primers were added to 12.5 μL PCR master mix (final volume of 25 μL). An initial denaturation at 94°C for 3 min was followed by 35 cycles of denaturation at 94°C for 30 sec, annealing at 60°C for 30 sec, and extension at 72°C for 45 sec followed by a final extension step at 72°C for 10 min. Eventually, the PCR product was run on a 2% agarose gel, the 168 bp band was interpreted as a positive result, and the 218 bp band was considered as the internal control (10).

Data were analyzed using SPSS 17 (SPSS Inc., Chicago, Il., USA). Chi-square (X^2^) was used to determine the association between qualitative variables like mutations of JAK2, MPL, CALR, and type of myeloproliferative neoplasm and sex. ANOVA test was utilized to analyze the difference between quantitative variables like leukocytosis, hemoglobin level, and platelet count in different mutation categories. P-value<0.05 was considered as a significant level.

## Results

**Figure. 1:**
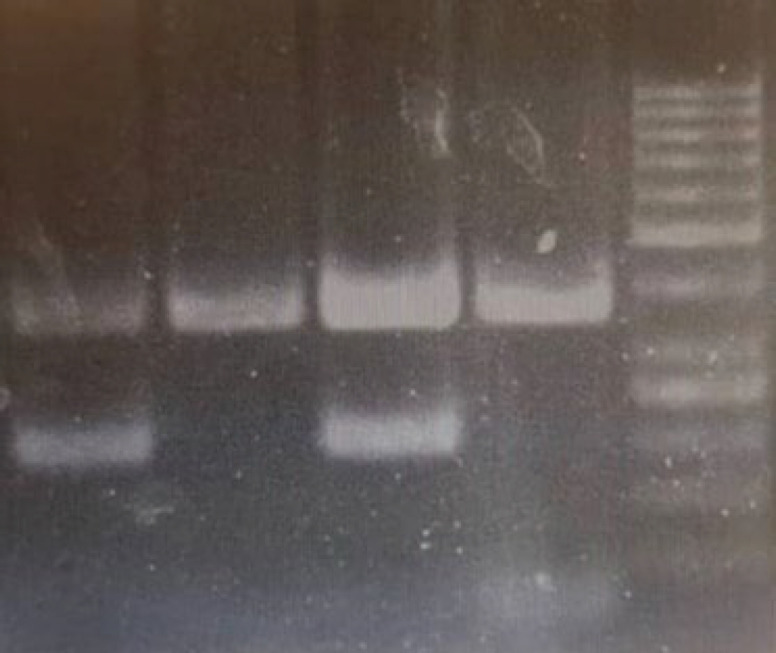
Electrophoresis of JAK2 PCR products. Positive JAK2 V617F mutations in lanes 1 and 3 with a PCR product of 203 bp

**Figure. 2:**
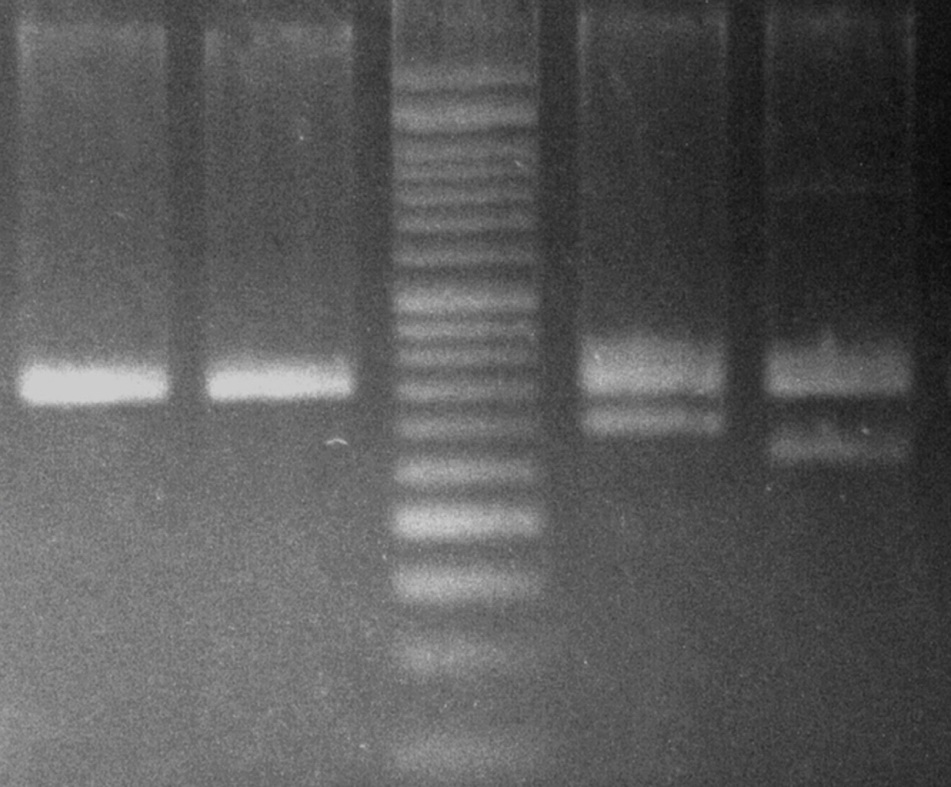
Electrophoresis of CALR PCR products. Positive CALR type 1 and type 2 in lanes 4 and 5 with PCR products of 302 and 272 bp respectively

Seventy-one patients with BCR-ABL negative MPNs were evaluated in this study from 2018 to 2019. Thirty-eight subjects were male and the remaining thirty-one were female. They had an age mean of 53.11±16.03 years (ranging from 23 to 88 years). Twenty-three patients were diagnosed as PV of which 21 cases (91.3%) were reported to have positive JAK2 V617F mutation, and no mutation (triple-negative) was observed in two cases (8.7%) (Figure 1). Thirty-eight patients were classified as ET who had JAK2 V617F mutation, CALR type 1 mutation, CALR type 2 mutation, and no mutation (triple-negative) in 20 (52.6%), 7 (18.4%), 3 (7.9%), and 8 cases (21.1%) respectively (Figure 2). PMF was recognized in seven patients and showed JAK2 V617F, CALR type 1, CALR type 2, and no mutation (triple-negative) in 4 (57.1%), 1 (14.3%), 1(14.3%), and 1 (14.3%) respectively. The remaining three patients were categorized as MPN-U that showed JAK2 V617F mutation just in one patient (33.3%) and demonstrated no mutation (triple-negative) in three (66.6%). Surprisingly, none of the cases harbored MPL mutation in this study.

The patients' age with JAK2 V617F mutation (59.15±13.10) was significantly higher compared to those with CALR type 2 mutation (29.33±8.50) (*P*=0.03), and patients with no mutation (41.92±14.77) (p=0.001), but this difference did not reach a significant level between JAK2 V617F (59.15±13.10) and CALR type 1 (46±16.08) (*P*=0.091).

Complete blood count parameters including platelet count, neutrophil percent, and lymphocyte percent also showed a significant difference between various types of mutations (Table 1). Platelet count was remarkably higher in patients with CALR type 1 mutation. White blood cell count difference did not reach a significant level between mutation groups, but differential count showed a significant difference in such a way that neutrophil percent was higher in JAK2 V617F mutation (70.78±10.14%) compared to CALR type 2 mutation (53.47±0.96%), and triple-negative cases (58.80±13.02). The lymphocyte percent was also reciprocally lower in JAK2 V617F mutation (19.01±8.61%) in comparison with CALR type 2 mutation (37.36±1.80), and triple-negative cases (31.28±10.95). 

**Table 1:** Complete blood count parameters in BCR-ABL negative MPNs with JAK2, CALR, and triple-negative mutations

	JAK2 V617F	CALR type 1	CALR type 2	Triple-negative	P-value
WBC (mean±SD /µL)	11419±5148	8375±2532	6700±2170	9228±3889	**0.141**
PMN (mean±SD %)	70.78±10.14	67.43±8.77	53.47±0.96	59.80±13.02	0.003
LYMPH (mean±SD %)	19.01±8.61	23.46±7.24	37.36±1.80	31.28±10.95	0.000
MXD (mean±SD %)	9.23±6.86	9.31±2.17	9.36±2.82	9.16±3.87	**1.000**
Hb (mean±SD g/dL)	15.51±3.73	14.16±3.13	13.46±1.45	13.90±3.22	**0.367**
Plt (mean±SD /µL)	613210± 304864	1240400± 402053	967000± 617075	723500± 264304	0.000

Abbreviation: WBC= white blood cells, PMN= polymorphonulcear cells, LYMP= lymphocytes, MXD= mixed cells (monocyte, eosinophil, and immature myeloid cells), Hb= hemoglobin, Plt= platelets.

## Discussion

There was a slight male predominance in this study (male to female ratio= 1.22:1). JAK2 V617F was the only mutation in PV which was detected in more than ninety percent of the cases. JAK2 V617F mutation was also the most prevalent mutation in other MPNs like ET and PMF followed by CALR mutations. Surprisingly, no MPL mutation was detected in any of the cases. All these mutations were mutually exclusive and none of them co-occurred. Those with JAK2 V617F mutations had significantly higher age and neutrophil percent. CALR was associated with significantly higher platelet count and lymphocyte percent. Although leukocyte count and hemoglobin levels were higher in patients with JAK2 V617F mutation compared to CALR, this difference did not reach a significant level statistically. 

In a study by Kim* et al.*, none of the PV patients showed a CALR mutation, while JAK2 was seen in 91.4% of PV patients. CALR mutation was seen in 14.8% of PMF patients and 17.7% of ET patients, compared with 63.4% and 63.3% for JAK2, respectively. Approximately, 37.5% of MPN-U patients showed CALR mutation which was higher than the PMF and ET ratio of CALR to JAK2. The results of their study which was conducted in Korea were similar to the Western population. In Kim* et al.* study, CALR was the most common mutation detected in patients with MPN-U. In this study, they concluded that the clinical outcome of CALR mutation varies among different types of MPN and still needs further investigation (11). Their findings were relatively similar to the current study except for the JAK2 V617F as the prevalent mutation in MPN-U patients in the current study. In a recent retrospective study on 172 subjects in China by Lin* et al.* the frequency of JAK2 V617F was the same as the current study in PV and was 91.3%. In Lin* et al.* study, JAK2 V617F mutation was approximately 10% higher and CALR mutation was roughly 10% lower in ET and PMF compared with the current study. This difference might be justified considering the methodological differences in molecular assays and different ethnicity and environmental factors (12). 

CALR mutations are found in approximately 20-25% of ET and PMF patients but are not seen in PV patients. Most CALR mutations are deletions or insertions to exon 9 that cause frameshift mutations. The type 1 mutation, which results in a 52 bp deletion, is seen in approximately 50% of patients with CALR mutation, and the type 2 mutation, which results from the insertions of 5 bp TTGTC, is responsible for approximately 30% of CALR mutations (7, 13-15). In patients with ET, CALR mutation was associated with low Hb, lower leukocyte counts, and higher platelets (15-17).

In the comparison made by Lin* et al.*, the mutation rates of MPL exon 10 and CALR exon 9 were similar to other studies, but the JAK2 V617F mutation showed a difference of 65 to 98%, which may be due to racial differences or different sensitivities of the detection methods. In this study, those patients who had the CALR exon 9 mutation were younger than those who had the JAK2 V617F mutation in ET and PMF. Besides, people who did not have any mutations were the youngest (18). In the current study, those patients with CALR type 2 mutation were the youngest. Thus CALR mutation study should be considered as one of the first diagnostic tests in young MPN patients with remarkably high platelet counts (approximately > 1000000/µL). 

MPL mutation was not found in this study which may indicate its low incidence in the southwest of Iran and therefore it should not be considered as a first-line molecular diagnostic test in this region. A recent systematic review and meta-analysis also showed that MPL mutation frequency was 0% in PV, 0.9 to 12.4% in ET, and 0 to 17% in PMF in various studies from different regions of the World (19).

Studies by Klampfl* et al.* and Nangalia* et al.* have shown that JAK2, MPL or CALR mutations are mutually exclusive and do not occur together as there (6-7). However, the co-occurrence of these mutations has been recently reported (20-21). The current study did not show the co-occurrence of these mutations either.

One of the drawbacks of this study is the lack of evaluation of JAK2 exon 12 and sequencing of CALR gene which might help to detect more mutations and reduce the number of triple-negative cases in the current study.

## Conclusion

The frequency of JAK2 and CALR was relatively similar to previous studies in the literature, but MPL mutation was not found in the current study and its frequency seems to be lower than what is expected. JAK2 mutation was associated with higher age and neutrophil percent. CALR mutation was associated with a higher platelet count. 
